# Supplementary material for: Edge-based graph neural network for ranking critical road segments in a network
Source: PLoS One. 2023 Dec 21;18(12):e0296045. doi: 10.1371/journal.pone.0296045 (PMC10734987; doi:10.1371/journal.pone.0296045)
Supplement: Attachment — Submitted filename: Response to Reviewers.pdf [file pone.0296045.s001.pdf]

We thank the editors and the reviewers for handling and reviewing the manuscript for technical merits. We have responded to all the questions and comments raised during the technical round.

Response to Comments from Academic Editor

Editor: Journal requirements: When submitting your revision, we need you to address these additional requirements.

Editor: 1) Please ensure that your manuscript meets PLOS ONE's style requirements, including those for file naming. The PLOS ONE style templates can be found at:
https://journals.plos.org/plosone/s/file?id=wjVg/PLOSOne_formatting_sample_main_body.pdf
https://journals.plos.org/plosone/s/file?id=ba62/PLOSOne_formatting_sample_title_authors_affiliations.pdf

Response: We have adjusted the authors' affiliations and legends to meet PLOS ONE style format. Additionally, changes have been made to the file naming of the figures.

Editor: 2) Please note that PLOS ONE has specific guidelines on code sharing for submissions in which author-generated code underpins the findings in the manuscript. In these cases, all author-generated code must be made available without restrictions upon publication of the work. Please review our guidelines at:
https://journals.plos.org/plosone/s/materials-and-software-sharing#loc-sharing-code
and ensure that your code is shared in a way that follows best practices and facilitates reproducibility and reuse.

Response: We will provide the code with a DOI (Digital Object Identifier) upon acceptance of the paper. The Data Availability Statement is added in the manuscript as follows:

Data for the U.S. system is obtained from the Environmental Systems Research Institute, Inc. (ESRI) [1] https://hub.arcgis.com/datasets/esri::usa-freeway-system/explore?layer=1. The network information of Minnesota state road network is obtained from the network repository [2] https://networkrepository.com. The remaining four transportation network datasets (Aachen, Edinburgh, Luxembourg, and Santa Barbara Transportation Networks) are acquired from Pyrosm, a Python library for OpenStreetMap data [3] https://doi.org/10.5281/zenodo.4279527. The edge weights are assigned from travel times for all these transportation network and are obtained through the Google Distance Matrix API [4] https://developers.google.com/maps/documentation/distance-matrix/overview.

Editor: 3) We note that the grant information you provided in the 'Funding Information' and 'Financial Disclosure' sections do not match. When you resubmit, please provide the correct grant numbers for the awards you received for your study in the 'Funding Information' section.

Response: We have now provided this below to match the two sections. And we have added the following statement in the cover letter.

The research award (Award No. 20220480) was received by SN and ET from the funding agency Los Angeles Bureau of Engineering (https://engineering.lacity.gov/). The funders had no role in study design, data collection, and analysis, the decision to publish, or preparation of the

manuscript.

Editor: 4) Thank you for stating the following in the Acknowledgments Section of your manuscript:

"We gratefully acknowledge the funding support provided by the City of Los Angeles's Bureau of Engineering (BOE)."

We note that you have provided funding information that is not currently declared in your Funding Statement. However, funding information should not appear in the Acknowledgments section or other areas of your manuscript. We will only publish funding information present in the Funding Statement section of the online submission form.

Please remove any funding-related text from the manuscript and let us know how you would like to update your Funding Statement. Currently, your Funding Statement reads as follows:

"The funders had no role in study design, data collection and analysis, decision to publish, or preparation of the manuscript."

"Please include your amended statements within your cover letter; we will change the online submission form on your behalf."

Response: We have deleted the funding information-related statement from the Acknowledgements, have modified the funding statement, and included it in the cover letter.

Editor: 5) We note that you have stated that you will provide repository information for your data at acceptance. Should your manuscript be accepted for publication, we will hold it until you provide the relevant accession numbers or DOIs necessary to access your data. If you wish to make changes to your Data Availability statement, please describe these changes in your cover letter and we will update your Data Availability statement to reflect the information you provide.

Response: We will share the DOI including the code once our paper is accepted in the journal.

Editor: 6) We note that Figures 13 and 15 in your submission contain [map/satellite] images which may be copyrighted. All PLOS content is published under the Creative Commons Attribution License (CC BY 4.0), which means that the manuscript, images, and Supporting Information files will be freely available online, and any third party is permitted to access, download, copy, distribute, and use these materials in any way, even commercially, with proper attribution. For these reasons, we cannot publish previously copyrighted maps or satellite images created using proprietary data, such as Google software (Google Maps, Street View, and Earth). For more information, see our copyright guidelines:

http://journals.plos.org/plosone/s/licenses-and-copyright

We require you to either (1) present written permission from the copyright holder to publish these figures specifically under the CC BY 4.0 license, or (2) remove the figures from your submission:

a. You may seek permission from the original copyright holder of Figures 13 and 15 to publish the content specifically under the CC BY 4.0 license. We recommend that you contact the original copyright holder with the Content Permission Form (http://journals.plos.org/plosone/s/file?id=7c09/content-permission-form.pdf) and the following text:

"I request permission for the open-access journal PLOS ONE to publish XXX under the Creative Commons Attribution License (CCAL) CC BY 4.0 (http://creativecommons.org/licenses/by/

4.0/). Please be aware that this license allows unrestricted use and distribution, even commercially, by third parties. Please reply and provide explicit written permission to publish XXX under a CC BY license and complete the attached form."

Please upload the completed Content Permission Form or other proof of granted permissions as an "Other" file with your submission.

In the figure caption of the copyrighted figure, please include the following text: “Reprinted from [ref] under a CC BY license, with permission from [name of publisher], original copyright [original copyright year]."

b. If you are unable to obtain permission from the original copyright holder to publish these figures under the CC BY 4.0 license or if the copyright holder’s requirements are incompatible with the CC BY 4.0 license, please either i) remove the figure or ii) supply a replacement figure that complies with the CC BY 4.0 license. Please check copyright information on all replacement figures and update the figure caption with source information. If applicable, please specify in the figure caption text when a figure is similar but not identical to the original image and is therefore for illustrative purposes only.

The following resources for replacing copyrighted map figures may be helpful:

USGS National Map Viewer (public domain):http://viewer.nationalmap.gov/viewer/

The Gateway to Astronaut Photography of Earth (public domain): http://eol.jsc.nasa.gov/sseop/clickmap/

Maps at the CIA (public domain):https://www.cia.gov/library/publications/the-world-factbook/index.htmlandhttps://www.cia.gov/library/publications/cia-maps-publications/index.html

NASA Earth Observatory (public domain): http://earthobservatory.nasa.gov/

Landsat: http://landsat.visibleearth.nasa.gov/

USGS EROS (Earth Resources Observatory and Science (EROS) Center) (public domain): http://eros.usgs.gov/#

Natural Earth (public domain): http://www.naturalearthdata.com/

Response: The Figures are all obtained from OpenStreetMap (https://www.openstreetmap.org/) which is an open source content and free to use under open-content license.

Response to Comments from Reviewer #1

Reviewer: This paper studies the task of ranking critical road segments for transportation networks. The authors pose the transportation network as a graph with roads as edges and intersections as nodes and deploy a Graph Neural Network (GNN) for the task.

Strength:
1. Ranking critical road segments is indeed a challenging and meaningful task for transportation networks. GNNs are suitable approaches for the task.
2. The idea of the paper is reasonable and easy to follow.
3. The paper presents the result on both synthetic and real-world graph data, experimental settings (i.e., hyper-parameters and complexity analysis) are provided.
4. Some applications of the proposed framework are presented.

Response: We thank the reviewer for their positive assessment, insightful comments, and constructive feedback. We have diligently addressed all of the questions and have provided our responses below.

Reviewer: Questions: 1. The paper generates 1000 training graphs, 100 validation graphs, and 100 test graphs. What's the difference with a single real-world road network (i.e., U.S. Freeway network), what are the train, valid and test dataset details of the real-world network?

Response: In our real-world network scenario, we generate training, testing, and validation datasets by modifying edge weights between nodes, following Case I and Case II protocols. These edge weights correspond to travel times obtained from the Google distance matrix API [4]. Minor congestion affects travel times, resulting in varying edge weights for each dataset. In Case I, we adjust only the edge weights, while in Case II, we modify both the number of edges and their weights within the graph. We have revised the following text as follows.

This following ablation study simulates the edge importance ranking due to a dynamic change in the network parameters resulting from construction activities or functional factors such as travel time. Two scenarios are considered in this study:

(i) Case I: In this case, the effects of minor congestion or interruption of traffic on roads are simulated through random perturbations in the edge weights according to $r \times \Omega_i$ where Ω_i is the weight of edge i, and the values of r are sampled from a uniform distribution $\mathscr{U}[0.8, 1.2]$. The number of nodes and edges for the graphs remains unchanged.

(ii) Case II: In this case, major interruption scenarios rendering a small number of edges inoperable, such as from accidents or natural disasters, are simulated. In addition to the edge weights specified previously, i.e., $r \times \Omega_i$ with $r \in \mathscr{U}[0.8, 1.2]$, the number of edges in the graph is modified as well. The number of edges is sampled from a discrete uniform distribution $\lfloor \mathscr{U}[0.99, 1] \times \mathscr{E} \rceil$ – which is based on a maximum of 1% edge deletion from the original network. Here, $\lfloor \cdot \rceil$ denotes the nearest integer function. While the 1% edge deletion number is arbitrarily assumed for the ablation study, this is not far from reports from previous events such as from a 20-year return period event resulting in 0.6%-0.7% loss in the road inventory [5].

We have also added the following sentence and the table caption to the revised manuscript for clarity.

In the context of GNN training, validation, and testing, the primary distinction among simulations lies in the change of edge weights, reflecting changes in travel time due to factors such as congestion (Case-I) and a deletion of edges (e.g., due to road closures) resulting from catastrophic events (Case-II). More details regarding this change can be found in Table 4.

Table 4: Generation parameters of the transportation networks; for each network, the training, testing, and validation data are generated from a range of edge weights as follows: $\Omega_i = 0.5 \times (\mathbb{G}_{\vec{i}} + \mathbb{G}_{\overleftarrow{i}}) \times \mathscr{U}[0.8, 1.2]$ for undirected graphs, and $\Omega_{\vec{i}} = \mathbb{G}_{\vec{i}} \times \mathscr{U}[0.8, 1.2]$ and $\Omega_{\overleftarrow{i}} = \mathbb{G}_{\overleftarrow{i}} \times \mathscr{U}[0.8, 1.2]$ for bidirected graphs.

Transportation Network	Generation Parameters	Case I	Case II
US Highway, USA	# nodes<br># edge	997<br>1490	997<br>$\lfloor \mathscr{U}[0.99, 1] \times 1490 \rceil$
Minnesota, USA	# nodes<br># edge	2640<br>3302	2640<br>$\lfloor \mathscr{U}[0.99, 1] \times 3302 \rceil$
Aachen, Germany	# nodes<br># edge	791<br>1498	791<br>$\lfloor \mathscr{U}[0.99, 1] \times 1498 \rceil$
Edinburgh, Scotland	# nodes<br># edge	864<br>1497	864<br>$\lfloor \mathscr{U}[0.99, 1] \times 1497 \rceil$
Luxembourg, Europe	# nodes<br># edge	692<br>1495	692<br>$\lfloor \mathscr{U}[0.99, 1] \times 1495 \rceil$
Santa Barbara USA	# nodes<br># edge	821<br>1499	821<br>$\lfloor \mathscr{U}[0.99, 1] \times 1499 \rceil$

Reviewer: Weakness: 1. It is better to present the detail of generated synthetic and real-world graph data in two tables.

Response: We agree, the details are now contained in two tables, Table 2 and Table 4.

Reviewer: 2. More experiments are needed like representation visualization to prove the effectiveness of the proposed framework.

Response: We have now added experiments on four new transportation networks: Aachen city transportation network (Germany), Edinburgh city transportation network (Scotland), Road network of country of Luxembourg (Europe), and Santa Barbara city transportation network (US). The details about these networks are shown in Table 4. We have included these results in the revised manuscript.

The Spearman's coefficient of our GNN-based framework on additional four worldwide networks are shown in Table 7. The average Spearman correlation exceeds 0.9, which underscores the performance of our approach.

Table 7: Spearman's Coefficient on Aachen, Edinburgh, Luxembourg, and Santa Barbara Transportation Networks

City	Graph	Case I		Case II	
		Undirected	Bidirected	Undirected	Bidirected
Aachen	Train	0.92 ± 0.007	0.92 ± 0.007	0.90 ± 0.011	0.91 ± 0.008
	Test	0.89 ± 0.021	0.89 ± 0.017	0.90 ± 0.010	0.90 ± 0.010
Edinburgh	Train	0.94 ± 0.005	0.95 ± 0.005	0.92 ± 0.009	0.92 ± 0.010
	Test	0.91 ± 0.012	0.92 ± 0.011	0.92 ± 0.010	0.92 ± 0.012
Luxembourg	Train	0.88 ± 0.008	0.89 ± 0.008	0.87 ± 0.009	0.87 ± 0.010
	Test	0.85 ± 0.014	0.85 ± 0.019	0.86 ± 0.011	0.86 ± 0.011
Santa Barbara	Train	0.93 ± 0.006	0.95 ± 0.004	0.92 ± 0.009	0.93 ± 0.009
	Test	0.92 ± 0.009	0.93 ± 0.007	0.92 ± 0.016	0.93 ± 0.011

Reviewer: 3. It is highly recommended for authors include more reviews about GNNs in related work [1-7] and compare more SOTA baselines to show the effectiveness of the provided method.

[1] Dynamic Hypergraph Structure Learning for Traffic Flow Forecasting. ICDE 2023

[2] Analysis of robustness of urban bus network. Chinese Physics B 2016

[3] A Comprehensive Survey on Deep Graph Representation Learning. arXiv 2023

[4] Kernel-based Substructure Exploration for Next POI Recommendation. ICDM 2022

[5] DisenHAN: Disentangled Heterogeneous Graph Attention Network for Recommendation. CIKM 2020

[6] DisenCite: Graph-based Disentangled Representation Learning for Context-Specific Citation Generation. AAAI 2022

[7] DisenCTR: Dynamic Graph-based Disentangled Representation for Click-Through Rate Prediction. SIGIR 2022

[8] HE-SNE: Heterogeneous Event Sequence-based Streaming Network Embedding for Dynamic Behaviors. IJCNN 2022

Response: We thank the reviewer for this comment. We will answer this in two parts as follows:

Regarding citing the reviewer recommended papers: We thank the reviewer for suggesting the list of papers. We have included Zhao et al. [6] as it uses GNN for traffic forecasting which is relevant to the transportation network application. We have also included Ju el al. [7] as it provides a detailed literature review on graph representation learning.

However, we find that the remaining papers are not directly relevant to this work as they address a bus network [8], recommendation system [9, 10], content-specific citation generation [11], click-through rate prediction [12], and capturing the evolving nature of user behaviors [13].

We have added the following sentences related to the works of Zhao et al. [6] and Ju el al. [7] in the revised paper.

Such learning-based methods have been used in transportation networks for travel time prediction [14], traffic forecasting [6], and missing traffic data imputation [15].

GNN is a deep learning architecture that leverages the graph structure and feature information to perform various tasks, including node/edge/graph classification [16, 7].

Regarding comparing SOTA baselines: The SOTA baseline methods can be categorized into two main groups: (a) utilitarian, which prioritizes overall efficiency or utility, and (b) egalitarian, which takes into account equity and population data while optimizing for efficiency. The utilitarian approach primarily relies on the topology of the transportation network, whereas the egalitarian approach incorporates both the origin-destination demand data and the network topology. In this paper, we have conducted a comparative analysis between our Graph Neural Network (GNN) based approach and several utilitarian methods, including (1) Edge betweenness Centrality, (2) Network efficiency-based vulnerability measure, and (3) Probabilistic measure of distance between networks. Based on computational efficiency, our GNN-based approach outperforms all the state-of-the-art methods. As part of our future work, we intend to integrate origin-destination demand data and equity-related information to compare our GNN based approach with egalitarian methods. Furthermore, it is worth noting that, to the best of our knowledge, while deep learning-based algorithms exist for approximating node importance ranking, there is currently no deep learning-based algorithm available for ranking the importance of edges or roads within the transportation network. This research endeavor was undertaken to address this gap. To address this in the revised manuscript, we have added the following sentences into the Conclusion section.

Additionally as a scope of future work, we intend to also integrate origin-destination demand data and equity-related information to compare our GNN based approach with egalitarian methods.

Response to Comments from Reviewer #2

Reviewer: This paper focuses on a interesting topic, which is identifying critical and vital road segments in a transportation network. The problem is solved via a transportation graph with roads as edges and intersections as nodes and deploy a GNN to rank the importance of road segments. The proposed model is evaluated on synthetic graphs and two real-world transportation networks. However, there exist certain areas in the paper that could be improved.

Response: We thank the reviewer for their insightful questions and comments. We have tried to thoroughly address all the questions and editorial comments and hope that the reviewer finds it to their satisfaction.

Reviewer: Firstly, the proposed method is evaluated on three synthetic but only two real-world datasets. Experiments on other real-world dataset are needed for more persuasive results.

Response: We have now included additional experiments on four transportation networks: Aachen city (Germany), Edinburgh city (Scotland), Luxembourg (Europe), and Santa Barbara (US). The details about these transportation networks are shown in Table 4.

Table 4: Generation parameters of the transportation networks: for each network, the training, testing, and validation data are generated from a range of edge weights as follows: $\Omega_i = 0.5 \times (\mathbb{G}_{\vec{i}} + \mathbb{G}_{\overleftarrow{i}}) \times \mathscr{U}[0.8, 1.2]$ for undirected graphs, and $\Omega_{\vec{i}} = \mathbb{G}_{\vec{i}} \times \mathscr{U}[0.8, 1.2]$ and $\Omega_{\overleftarrow{i}} = \mathbb{G}_{\overleftarrow{i}} \times \mathscr{U}[0.8, 1.2]$ for bidirected graphs.

Transportation Network	Generation Parameters	Case I	Case II
US Highway, USA	# nodes<br># edge	997<br>1490	997<br>$\lfloor \mathscr{U}[0.99, 1] \times 1490 \rceil$
Minnesota, USA	# nodes<br># edge	2640<br>3302	2640<br>$\lfloor \mathscr{U}[0.99, 1] \times 3302 \rceil$
Aachen, Germany	# nodes<br># edge	791<br>1498	791<br>$\lfloor \mathscr{U}[0.99, 1] \times 1498 \rceil$
Edinburgh, Scotland	# nodes<br># edge	864<br>1497	864<br>$\lfloor \mathscr{U}[0.99, 1] \times 1497 \rceil$
Luxembourg, Europe	# nodes<br># edge	692<br>1495	692<br>$\lfloor \mathscr{U}[0.99, 1] \times 1495 \rceil$
Santa Barbara USA	# nodes<br># edge	821<br>1499	821<br>$\lfloor \mathscr{U}[0.99, 1] \times 1499 \rceil$

We have modified the statements in the paper to reflect the addition to these new cases.

Table 7: Spearman's Coefficient on Aachen, Edinburgh, Luxembourg, and Santa Barbara Transportation Network

City	Graph	Case I		Case II	
		Undirected	Bidirected	Undirected	Bidirected
Aachen	Train	0.92 ± 0.007	0.92 ± 0.007	0.90 ± 0.011	0.91 ± 0.008
	Test	0.89 ± 0.021	0.89 ± 0.017	0.90 ± 0.010	0.90 ± 0.010
Edinburgh	Train	0.94 ± 0.005	0.95 ± 0.005	0.92 ± 0.009	0.92 ± 0.010
	Test	0.91 ± 0.012	0.92 ± 0.011	0.92 ± 0.010	0.92 ± 0.012
Luxembourg	Train	0.88 ± 0.008	0.89 ± 0.008	0.87 ± 0.009	0.87 ± 0.010
	Test	0.85 ± 0.014	0.85 ± 0.019	0.86 ± 0.011	0.86 ± 0.011
Santa Barbara	Train	0.93 ± 0.006	0.95 ± 0.004	0.92 ± 0.009	0.93 ± 0.009
	Test	0.92 ± 0.009	0.93 ± 0.007	0.92 ± 0.016	0.93 ± 0.011

Reviewer: Additionally, this paper lacks a detailed visualization of the transportation network and ranked segments to intuitively show the effect of applied method.

Response: In response to the reviewer's comment, we have now added a new figure to aid in visual presentation. We have made the necessary changes in the manuscript text to reflect this.

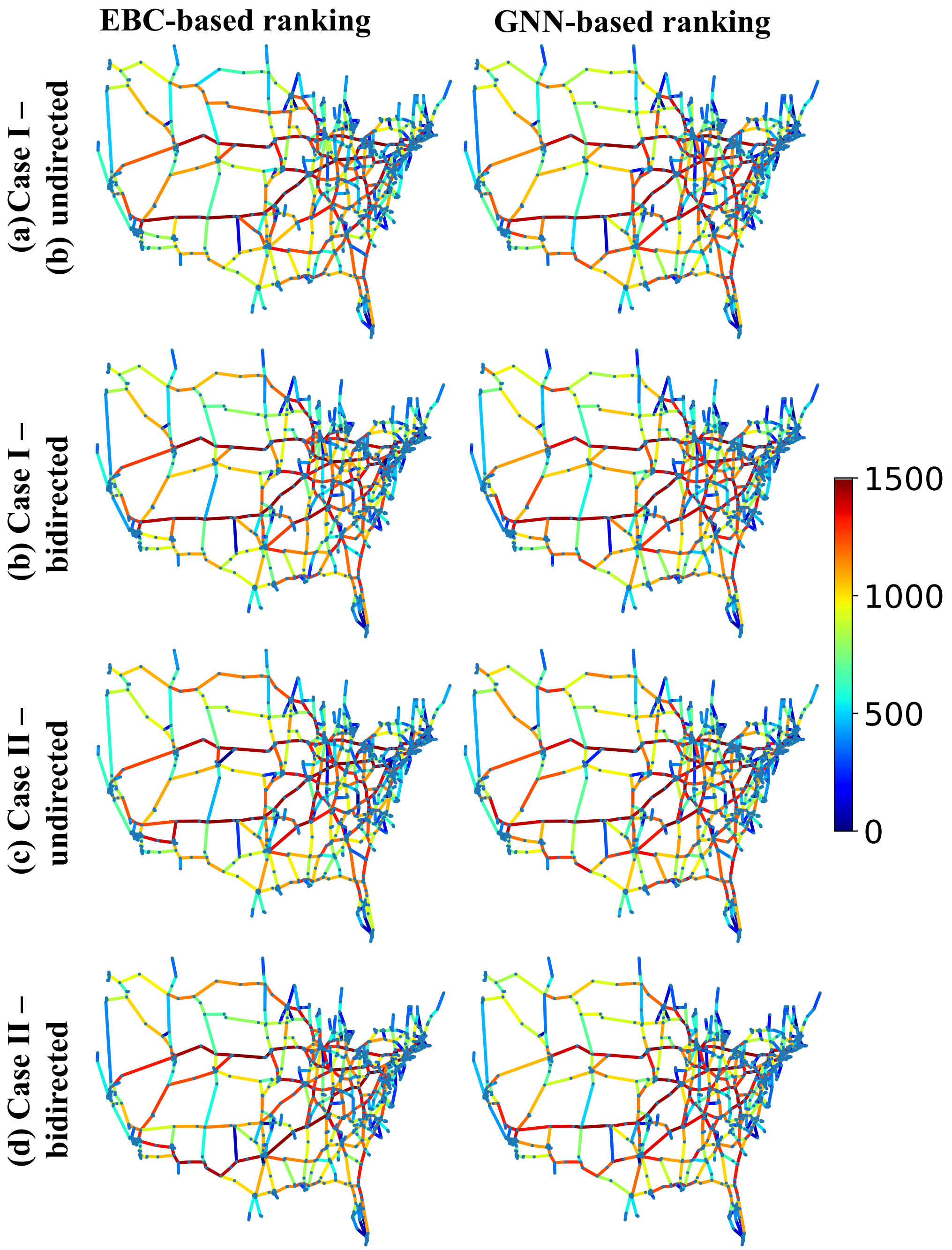

Figure 15: Comparison between EBC based road ranking and the proposed GNN-based road ranking of US highway network for different scenarios; the color bar shows the road ranking.

References

[1] E. Data and Maps. *USA Freeway System (over 1:500k)*. URL: https://hub.arcgis.com/datasets/esri::usa-freeway-system/explore?layer=1. (accessed: 04.04.2023).

[2] R. A. Rossi and N. K. Ahmed. "The Network Data Repository with Interactive Graph Analytics and Visualization". In: *AAAI*. 2015. URL: https://networkrepository.com.

[3] H. Tenkanen. *HTenkanen/pyrosm: v0.6.0*. Version v0.6.0. Nov. 2020. DOI: 10.5281/zenodo.4279527. URL: https://doi.org/10.5281/zenodo.4279527.

[4] Google. *Distance Matrix API*. Google, Nov. 25, 2022. URL: https://developers.google.com/maps/documentation/distance-matrix/overview.

[5] F. J. Shahdani et al. "Assessing Flood Indirect Impacts on Road Transport Networks Applying Mesoscopic Traffic Modelling: The Case Study of Santarém, Portugal". In: *Applied Sciences* 12.6 (2022), p. 3076.

[6] Y. Zhao et al. "Dynamic Hypergraph Structure Learning for Traffic Flow Forecasting". In: ICDE. 2023.

[7] W. Ju et al. "A Comprehensive Survey on Deep Graph Representation Learning". In: *arXiv preprint arXiv:2304.05055* (2023).

[8] T. Ren et al. "Analysis of robustness of urban bus network". In: *Chinese Physics B* 25.2 (2016), p. 020101.

[9] W. Ju et al. "Kernel-based substructure exploration for next POI recommendation". In: *2022 IEEE International Conference on Data Mining (ICDM)*. IEEE. 2022, pp. 221–230.

[10] Y. Wang et al. "Disenhan: Disentangled heterogeneous graph attention network for recommendation". In: *Proceedings of the 29th ACM international conference on information & knowledge management*. 2020, pp. 1605–1614.

[11] Y. Wang et al. "DisenCite: Graph-Based Disentangled Representation Learning for Context-Specific Citation Generation". In: *Proceedings of the AAAI Conference on Artificial Intelligence*. Vol. 36. 10. 2022, pp. 11449–11458.

[12] Y. Wang et al. "DisenCTR: Dynamic graph-based disentangled representation for click-through rate prediction". In: *Proceedings of the 45th International ACM SIGIR Conference on Research and Development in Information Retrieval*. 2022, pp. 2314–2318.

[13] Y. Wang et al. "HE-SNE: Heterogeneous Event Sequence-based Streaming Network Embedding for Dynamic Behaviors". In: *2022 International Joint Conference on Neural Networks (IJCNN)*. IEEE. 2022, pp. 1–8.

[14] J.-u.-R. Chughtai, I. U. Haq, and M. Muneeb. "An attention-based recurrent learning model for short-term travel time prediction". In: *Plos one* 17.12 (2022), e0278064.

[15] X. Kong et al. "Dynamic graph convolutional recurrent imputation network for spatiotemporal traffic missing data". In: *Knowledge-Based Systems* 261 (2023), p. 110188.

[16] J. Zhou et al. "Graph neural networks: A review of methods and applications". In: *AI Open* 1 (2020), pp. 57–81.
