# Supplementary material for: Edge-based graph neural network for ranking critical road segments in a network
Source: PLoS One. 2023 Dec 21;18(12):e0296045. doi: 10.1371/journal.pone.0296045 (PMC10734987; doi:10.1371/journal.pone.0296045)
Supplement: Attachment — Submitted filename: Response to Reviewers.pdf [file pone.0296045.s002.pdf]

We thank the editors and the reviewers for handling and reviewing the manuscript for technical merits. We have responded to all the questions and comments raised during the technical round.

Response to Comments from Reviewer #4

Reviewer: The authors proposed a GNN-based system to calculate the edge betweenness certainty in transportation networks. They have explained the methodology in detail, accompanied by proper diagrams and algorithms, in a clear and comprehensive manner. Additionally, the authors conducted evaluations on both synthetic and real-world data, presenting the results in detail. However, several issues have been identified in the manuscript, as follows:

Response: We thank the reviewer for their insightful questions and comments. We have tried to thoroughly address all the questions and editorial comments and hope that the reviewer finds it to their satisfaction.

Reviewer: 1. The authors are advised to include a paragraph about road network vulnerability analysis and its importance. Although, the introduction paragraph of the manuscript covers certain aspects insufficiently, making it challenging for a general reader to grasp the significance of this work.

Response: We have included the following paragraph in the introduction section to address this comment:

Road network vulnerability analysis is a crucial process that involves assessing the susceptibility of a transportation infrastructure to various risks and potential disruptions. This analysis plays a pivotal role in enhancing the resilience of road networks, ensuring their ability to withstand and recover from unforeseen events such as natural disasters, accidents, or deliberate attacks. By systematically evaluating the vulnerabilities within a road network, transportation authorities can identify weak points, assess potential impact scenarios, and develop effective strategies to mitigate risks. The importance of road network vulnerability analysis lies in its ability to inform decision-makers about potential vulnerabilities, allowing for the implementation of targeted measures to enhance the overall reliability and functionality of the transportation system. This proactive approach not only helps minimize the impact of disruptions on traffic flow and public safety but also contributes to the sustainable development of resilient and adaptive transportation networks in the face of evolving challenges.

Reviewer: 2. The proposed GNN model is trained for only 50 epochs. However, according to Fig. 10(a), the training loss is steeply decreasing with each epoch. Therefore, if the model is trained for more epochs, it can optimize well and perform better. The authors are advised to train their model for more epochs and assess its performance.

Response: We have included the following paragraph in the 'Model training time' section to address this comment:

Fig 10(a) displays the evolution of the evaluated training and validation marginal ranking loss for each epoch. The progression of Spearman's rank correlation coefficient over epochs for both training and validation data is also depicted in Fig 10(b). It is observed from Fig 10(a) that after 50 epochs, the training loss continues to decrease while the validation loss starts to increase —

indicating the 'overfitting' phenomenon in deep learning [1]. As 'early stopping' is considered one of the robust ways to prevent overfitting by halting training when the validation loss ceases to improve, the training process was terminated at 50 epochs.

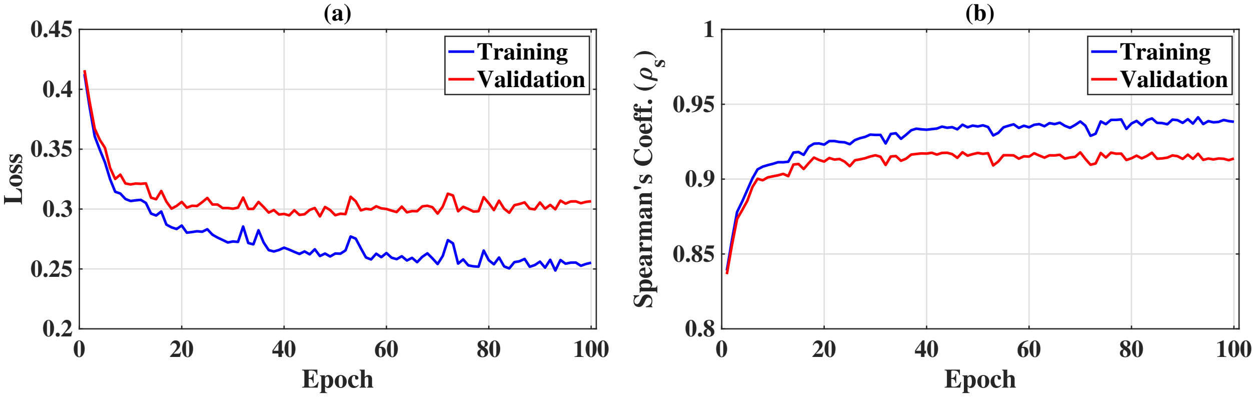

Figure 10: Evolution in learning phase – (a) Training and validation loss over epochs and (b) Evaluation metric (for training and validation data) over epochs.

Reviewer: 3. In the training phase of the proposed approach, the desired output was calculated by utilizing the conventional method for determining edge betweenness certainty (EBC). However, in Fig. 16, a comparison is made between conventional EBC-based ranking and the ranking obtained through the GNN. The authors need to justify this contradiction in detail.

Response: We have included the following paragraph in the 'Ablation studies on road network' section to address this comment:

For better visualization purposes, we compare the ranking of edges/roads using our proposed GNN with EBC, as depicted in Fig 15. This comparison plot demonstrates that the trained GNN architecture, using EBC-based ranking as a surrogate target, effectively mimics the performance of EBC-based ranking with much less computational cost, leveraging its capacity to generalize from a broad range of network parameter changes and disruption events. Hence, GNN-based ranking is useful for a fast approximation of critical road segments (Fig. 12), making it beneficial for the application of hazard simulation and rapid decision-making in emergency situations for large networks. It is also noteworthy that while the GNN was trained on EBC-based ranking, it has not been explicitly trained on network-efficiency-based vulnerability measure-based ranking (Eff. Vul.) and the vulnerability based on the probabilistic distance measure between networks based ranking (Prob. Vul.). Despite this, Fig 14 and Table 5 demonstrate that the GNN is capable of approximating the ranking based on these more computationally intensive quantities. This finding underscores the versatility of our proposed method and its potential applicability in scenarios where directly calculating vulnerability metrics might be impractical.

References

[1] I. Goodfellow, Y. Bengio, and A. Courville. *Deep learning*. MIT press, 2016.
